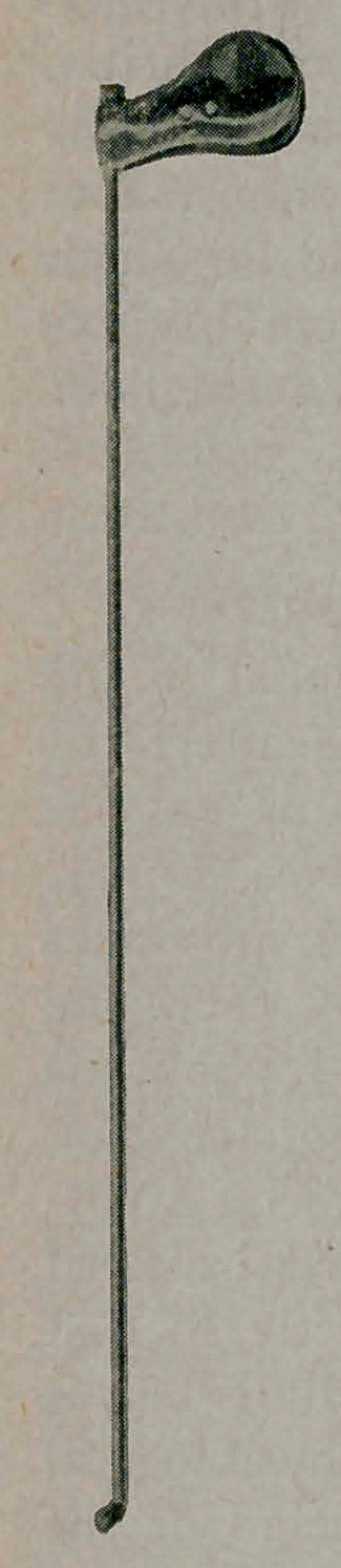# A New Urethroscopic Forceps

**Published:** 1910-12

**Authors:** Charles W. Bethune

**Affiliations:** Buffalo, N. Y.


### NEW INSTRUMENT

### A New Urethroscopic Forceps.

By CHARLES W. BETHUNE M.D., Buffalo, N. Y.

There are two difficulties encountered in using an alligator forceps through the urethroscope. In the first place a large tube must be used; in the second the shaft of the forceps is in the line of vision preventing one from seeing when the foreign body is between the blades.

To overcome these difficulties I designed the forceps shown here. The shaft has a caliber of 5 French and is 20 cm. in length, the blades are set at an agle of 90° to the shaft. This enables one to grasp a broken filliform, hat pin, or wad of cotton under the guidance of the eye instead of fishing around for it as must be done with an alligator forcep. The forceps can be introduced through and the blades separated to their fullest extent within the lumen of a 22F urethroscope.

The shaft is long enough to be used through a female cystoscope for removing foreign bodies or to bite off specimens for microscopic examination. I wish to acknowledge the care that the Electro Surgical Instrument Co. of Rochester, N. Y., has taken in making this instrument for me.

262 Niagara Street.

## Figures and Tables

**Figure f1:**